# Combined Diagnostic Accuracy of Total Leukocyte Count, Neutrophil Count, and Ultrasonography for the Diagnosis of Acute Appendicitis

**DOI:** 10.7759/cureus.13086

**Published:** 2021-02-02

**Authors:** Shehzadi Rimsha Fatima, Farhan Zaheer, Foad Ali Moosa, Shehanshah Muhammed Arqam, Raja Muhammad Mussab, Muhammad Saad Choudhry

**Affiliations:** 1 General Surgery, Civil Hospital Karachi, Dow University of Health Sciences, Karachi, PAK; 2 General Surgery, Jinnah Postgraduate Medical Centre, Karachi, PAK

**Keywords:** acute appendicitis, total leukocyte count, neutrophil count, ultrasound, surgical emergency, clinical diagnosis

## Abstract

Acute appendicitis is a common surgical emergency that classically presents with right lower abdominal pain and tenderness on palpation. The diagnosis is often based on clinical examination in order to avoid the complications of surgery delay, yielding a high rate of negative appendectomies. Ultrasonography is a regularly used modality for establishing the diagnosis, whereas abdominal computed tomography (CT) is often used in sonologically equivocal cases. Other parameters include total leukocyte count, granulocytes, C-reactive protein (CRP), leukocyte elastase activity, D-lactate, phospholipase A2, and interleukin-6 (IL-6). We conducted a prospective study to assess the combined accuracy of total leukocyte count, neutrophil count, and ultrasound as an integrated diagnostic tool. The results of these investigations were tabulated and compared to histopathological evidence of acute appendicitis on biopsy (taken as the gold standard) to calculate sensitivity, specificity, positive predictive value, and negative predictive value. Combined sensitivity and specificity were calculated using cross-tabulation, whereas diagnostic accuracy was estimated from the receiver operating curve (ROC) at the optimal cut-off point. The results showed that the absence of inflammatory findings on ultrasound and normal blood parameters (total leukocyte count and neutrophil count) have a high combined diagnostic accuracy and appendicitis may be ruled out.

## Introduction

Acute appendicitis is one of the most common surgical emergencies encountered by surgeons [1]. A typical patient having acute appendicitis presents with initial periumbilical pain that later shifts to the right lower abdomen and exhibits tenderness in the right iliac fossa on palpation. The accuracy of diagnosis based on clinical examination ranges between 76% and 92%. However, atypical symptoms like colicky abdominal pain, burning micturition, and diarrhea are common and can create difficulty in the establishment of a clinical diagnosis. Early accurate diagnosis in patients who require immediate surgical intervention is very crucial, as a delay can increase the rate of complications [2-3].

General surgeons have had a low threshold for appendectomy based on the clinical diagnosis in order to avoid delay and prevent complications, leading to a higher rate of negative appendectomies. This rate is found to be high (10%-44%), even in tertiary care centers with proper diagnostic facilities [4]. Negative appendectomies not only cause physical and emotional stress to patients but also cause a financial burden to the patients and public sector hospitals and increase the physical burden of hospital staff working in hectic emergency situations. This rate can be reduced with the help of tools such as focused abdominal computed tomography scan (FACT) for appendicitis. In a study conducted by Shahani et al., it was observed that FACT has a high sensitivity of 97.61%, specificity of 83.33%, and accuracy of 96.66% and can prove to be a good alternate for clinically and sonologically equivocal appendicitis [5]. Ali M et al. utilized computed tomography (CT) scans to differentiate perforated from non-perforated appendicitis and to decide on an operative versus non-operative treatment strategy [6]. However, the use of a CT scan as a routine diagnostic tool is not free from pitfalls like radiation hazards, non-availability in all hospitals, the financial cost to patients or hospitals, lack of guidelines, and indications for usage [7].

Ultrasound (U/S) is the most commonly used imaging tool in the emergency department for the diagnosis of acute appendicitis with a sensitivity of 83.7% and a specificity of 95.9%. It is non-invasive, portable, can be repeated frequently without the fear of radiation exposure, and is less expensive when compared with CT [8]. Researchers have conducted studies on various modalities to find suitable alternatives to CT and U/S. These include total leukocyte count (TLC), granulocytes, C-reactive protein (CRP), leukocyte elastase activity, D-lactate, phospholipase A2, and interleukin-6 (IL-6) [5-7]. Current medical literature displays a wide variation in evidence regarding the use of TLC and differential leukocyte count for the diagnosis of acute appendicitis [9-10]. A prospective study conducted by Ali et al. suggested that if investigations like TLC, neutrophil count, CRP, and U/S are not suggestive of acute appendicitis, it can be ruled out [11]. The aim of our study was to find out the combined diagnostic accuracy of TLC, neutrophil count, and U/S for the preoperative diagnosis of acute appendicitis, as it will help reduce the rate of negative appendectomies and improve the overall outcome of the patient.

## Materials and methods

This prospective study was conducted from November 2019 till May 2020. After receiving approval from the Institutional Review Board, consenting patients meeting inclusion criteria were enrolled in the study at the Department of Surgical Unit 1, Dr. Ruth K.M. Pfau Civil Hospital, Karachi. Patients from both genders were included. A detailed history of the illness and basic demographic information was taken at the time of admission, followed by a clinical examination for features of acute appendicitis. Those with a suspected clinical picture of acute appendicitis, having right lower abdominal pain, rebound tenderness in the right iliac fossa, anorexia, nausea, vomiting, and duration of symptoms within 72 hours were admitted. Exclusion criteria included patients who had an appendicular lump or an appendicular perforation (both assessed by clinical examination), pregnancy with appendicitis (assessed by relevant clinical history), previous history of urolithiasis or pelvic inflammatory disease, previous abdominal surgery or recent abdominal trauma, and failure to get consent.

Blood samples were drawn by the researcher in a disposable syringe under aseptic conditions for complete blood count (which revealed TLC and neutrophil count). TLC count of more than 11,000 cells/ml and neutrophil count of more than 75% were considered as supportive evidence in favor of acute appendicitis. After lab workup, an ultrasound abdomen was performed for the presence of acute appendicitis. U/S findings were considered positive if they showed a non-compressible, blind-ending, non-peristaltic bowel loop originating from the cecum (appendix), loculated para-cecal collection, and/or the finding of an appendicolith. The surgically excised specimens were sent for histopathology after being fixed in formalin 10% at the Department of Pathology, Dow Medical College, Civil Hospital, Karachi. A total of 177 samples were collected through the non-probability consecutive sampling technique.

The sample size was calculated by using TLC (sensitivity 76%, specificity 65%), the prevalence of acute appendicitis (50%), the margin of error (d = 10%), and a confidence interval of 95% using the Dr. Lin Naing sample size calculator for sensitivity and specificity. The Statistical Package for the Social Sciences (SPSS) version 23.0 (IBM Corp, Armonk, NY) was used for data collection and analysis. The study variables included age, gender, values of TLC and neutrophil count, U/S, and histopathological findings of the appendices. Histopathology reports were taken as the gold standard for diagnosis. Sensitivity, specificity, positive predictive value (PPV), and negative predictive value (NPV) were calculated for each test. Combined sensitivity and specificity were calculated using cross-tabulation. Diagnostic accuracy at the optimal cut-off threshold scores was derived from the receiver operating curve (ROC) for TLC, neutrophil count, and U/S.

## Results

The age range of patients included in our study was 13-60 years and the most prevalent age group in the sample population was found to be 21-30 years (38.8%). We had 122 (68.8%) male and 55 (31.2%) female patients. The histopathology reports of surgically removed appendices showed that 142 (80.22%) cases actually had acute appendicitis (Table 1).

**Table 1:** Descriptive statistics of age, gender, and histopathology * indicates histopathology reports that showed features consistent with acute appendicitis

		Frequency	Percentage (%)
Age	13-20	67	37.6
	21-30	69	38.8
	31-40	34	19.4
	41-50	7	2.4
	51-60	5	1.8
Gender	Male	122	68.8
	Female	55	31.2
Histopathology	Positive^*^	142	80.22
	Negative	35	19.78

**Figure 1:**
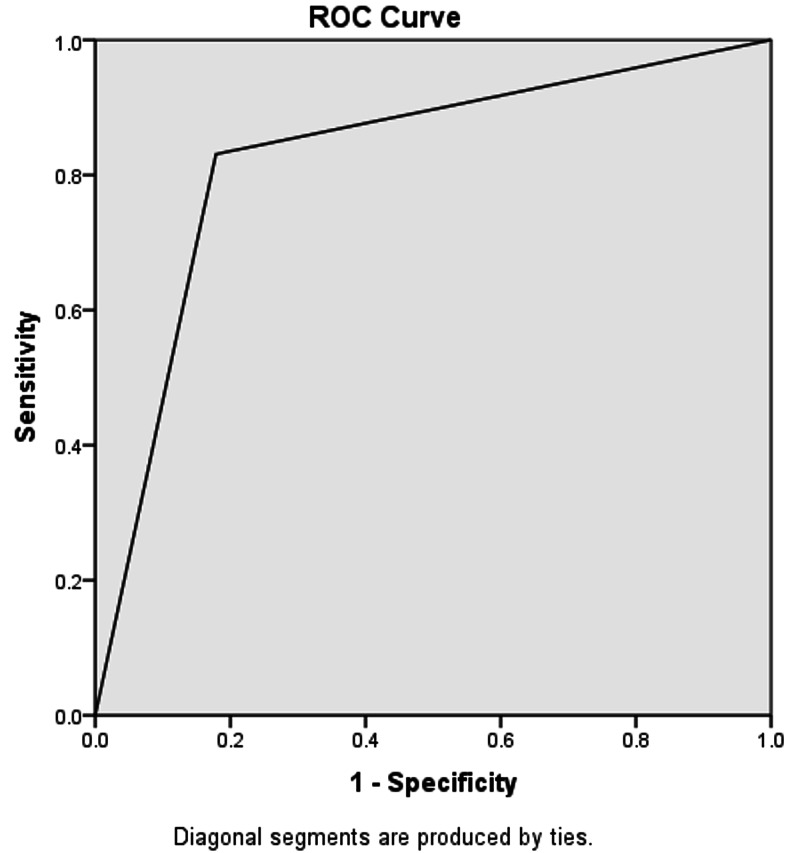
Area under the curve for TLC > 11,000/mm3 = 0.826 TLC: total leukocyte count

The histopathology report showed that 123 out of 128 patients who had TLC > 11,000/mm^3^ actually had appendicitis (true-positive). While 25 out of 49 patients who had evidence of acute appendicitis on histopathology were false-negative (TLC was < 11,000/mm^3^). The diagnostic accuracy of TLC > 11,000/mm^3^ was found to be 82.94%, sensitivity 83.10%, specificity 82.14%, PPV 95.93%, and NPV 48.94%. The area under the curve on ROC was 0.826 (refer to Figure 1 and Table 2).

**Table 2:** Describes the comparison of histopathological report findings with TLC, neutrophil count, and ultrasound findings TLC: total leukocyte count

		Histopathology		Total	Sensitivity	Specificity	Accuracy	Area Under Curve	Positive-Predictive Value (PPV)	Negative-Predictive Value (NPV)
		Acute Appendicitis	Normal Appendix							
TLC	Positive	123 (83.1%)	5 (17.9%)	128 (72.4%)	83.10%	82.14%	82.94%	0.826	95.93	48.94
	Negative	25 (16.9%)	24 (82.1%)	49 (27.6%)						
	Total	148 (100%)	29 (100%)	177 (100%)						
Neutrophils	Positive	130 (87.8%)	2 (6.9%)	132 (74.6%)	88.03	92.86%	88.82	0.904	98.43	60.47
	Negative	18 (12.1%)	27 (93.1%)	45 (25.4%)						
	Total	148 (100%)	29 (100%)	177 (100%)						
Ultrasound	Positive	127 (89.4%)	5 (17.9%)	132 (77.6%)	89.44%	82.14%	88.24	0.858	96.38	71.87
	Negative	15 (10.6 %)	23 (82.1%)	38 (22.4%)						
	Total	142 (100%)	28 (100%)	170 (100%)						

**Figure 2:**
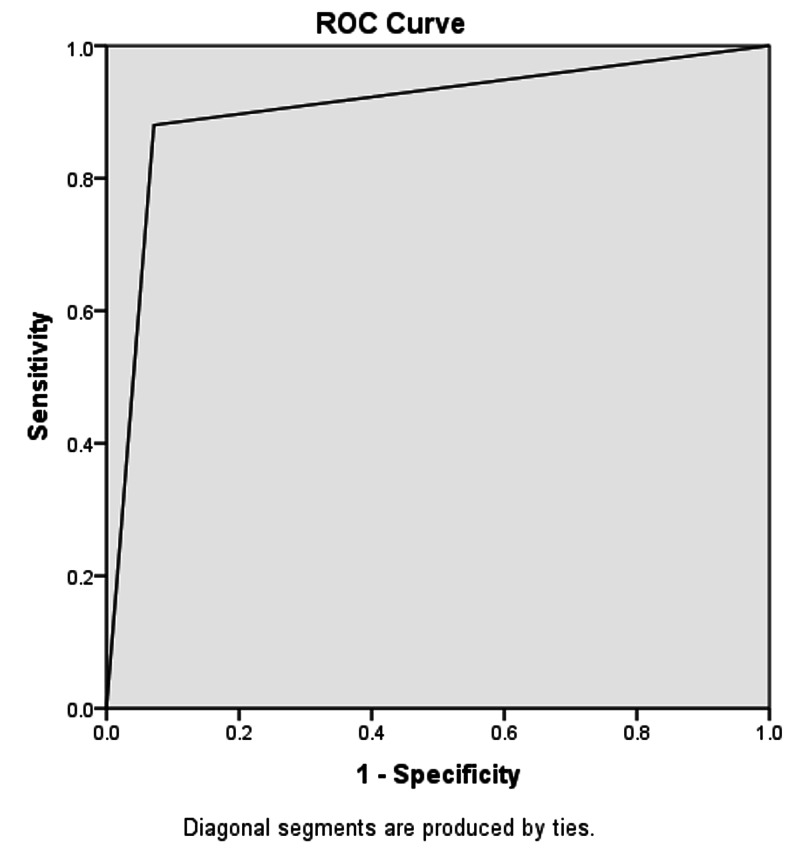
Area under the curve for neutrophil count > 75% = 0.904

On the other hand, 130 out of 132 patients who had neutrophil count > 75% were true-positive on the basis of histopathological findings, whereas 18 out of 45 patients who had evidence of acute appendicitis on histopathology were false-negative (the neutrophil count was < 75%). The accuracy of the neutrophil test in this study was calculated to be 88.82%, sensitivity 88.03%, specificity 92.86%, PPV 98.43%, and NPV 60.47%. The area under the curve for the neutrophil count on ROC was 0.904 (refer to Figure 2 and Table 2).

**Figure 3:**
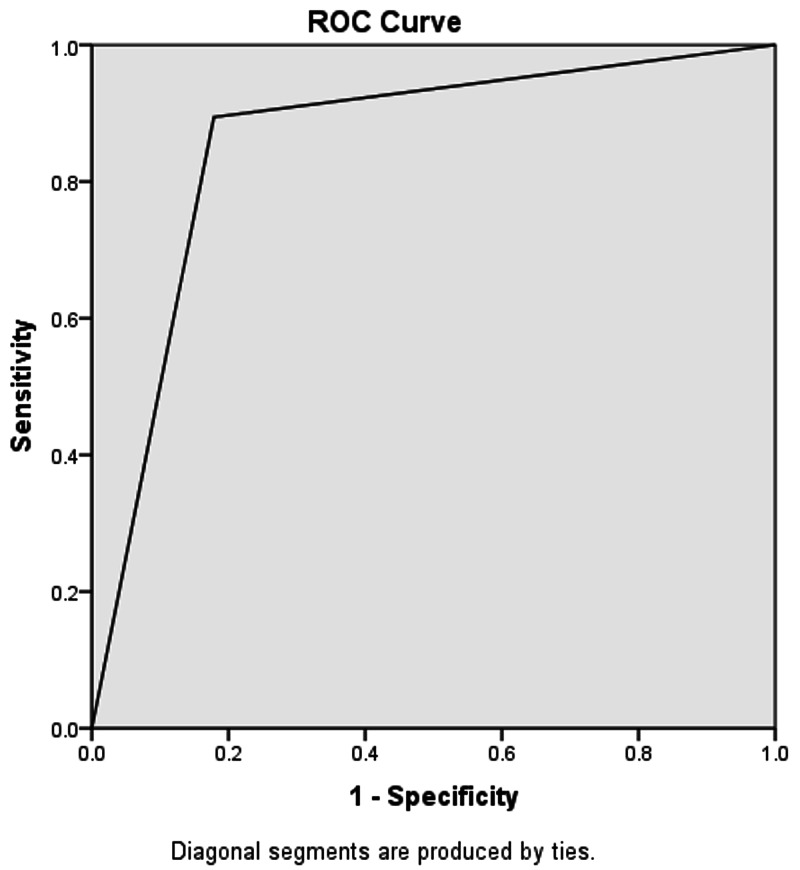
Area under the curve for the ultrasound scan = 0.858

For the U/S scan showing a non-compressible, blind-ending tubular structure and diameter of the appendix > 7mm. Out of 132 U/S positive patients, 127 were true-positive based on the histopathological report. In our study, five patients were false-positive, whereas 15 patients were false-negative for acute appendicitis on U/S examination. The accuracy of U/S was calculated to be 88.24%, sensitivity 89.44%, specificity 82.14%, PPV 96.38%, NPV 71.87%, and area under the curve 0.858 (refer to Figure 3 and Table 2). Possible reasons can be lymphoid hyperplasia, abdominal tuberculosis, salpingitis, or other causes of peritonitis, which may cause inflammatory changes around the appendix similar to acute appendicitis.

**Figure 4:**
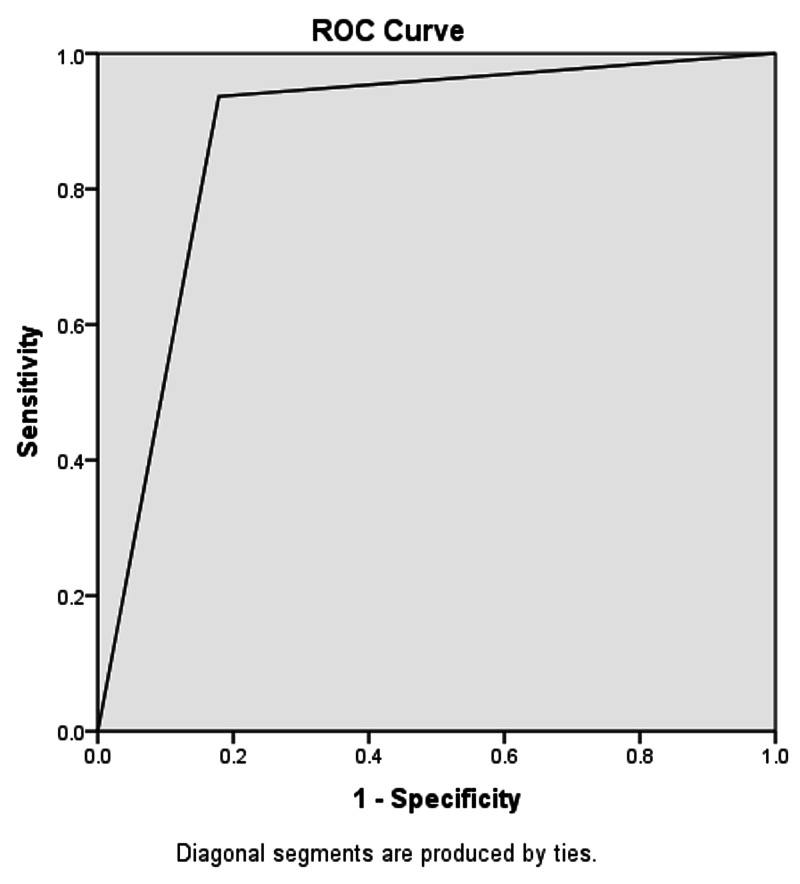
Area under the curve for combined TLC and neutrophil count = 0.879 TLC: total leukocyte count

All patients having TLC > 11,000/mm^3^ along with neutrophil count > 75% were combined into one group. Ninety-three point seven percent (93.7%) of these patients were diagnosed with appendicitis on the histopathology report. The accuracy of TLC and neutrophil count combined was found to be 88.24%, sensitivity 89.44%, specificity 82.14%, PPV 96.21%, NPV 60.53%, and area under the curve 0.879 (refer to Table 3 and Figure 4).

**Table 3:** Analysis of combined factors (cross-tabulated)

		Histopathology		Sensitivity	Specificity	Accuracy	Area Under Curve	Positive-Predictive Value (PPV)	Negative-Predictive Value (NPV)
		Acute Appendicitis	Normal Appendix						
TLC + Neutrophil Count	Positive	133 (93.7%)	5 (17.9%)	89.44%	82.14%	88.24%	0.879	96.21%	60.53%
	Negative	9 (6.3%)	23 (82.1%)						
TLC + Neutrophils Count + Ultrasonography	Positive	138 (97.2%)	5 (17.9%)	97.18%	82.14%	94.71%	0.897	96.50%	85.19%
	Negative	4 (2.8%)	23 (82.1%)						

**Figure 5:**
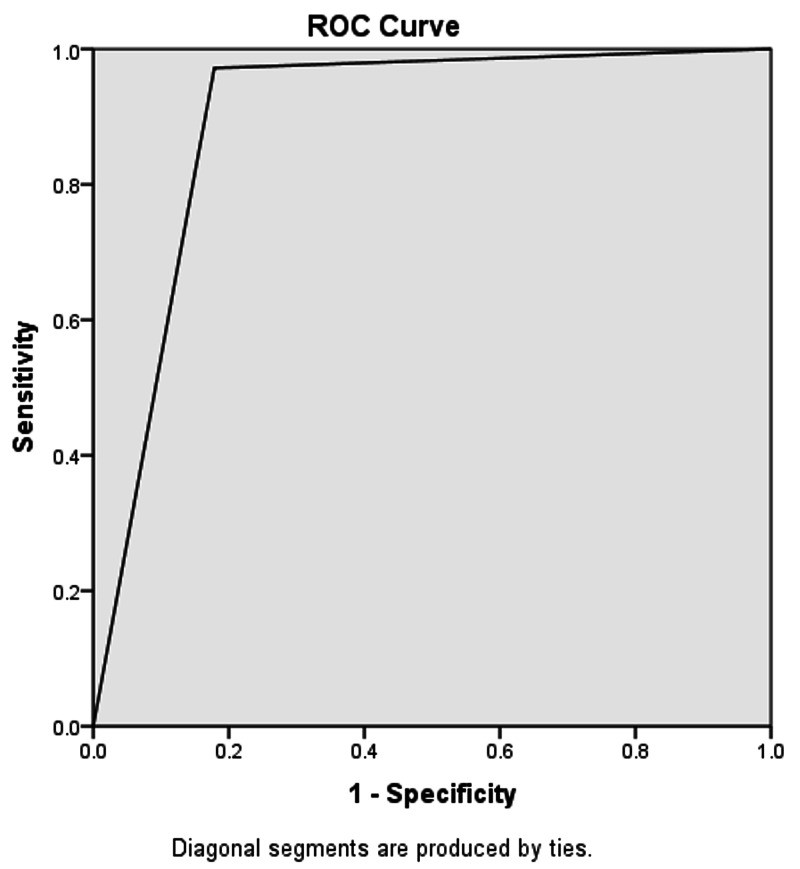
Area under the curve for combined TLC, neutrophil count, and U/S = 0.897 TLC: total leukocyte count; U/S: ultrasound

Likewise, patients having TLC > 11,000/mm^3^, neutrophil count > 75%, and ultrasonographic features showing non-compressible, blind-ending loop, and diameter of the appendix > 7 mm were combined into one group. Ninety-seven point two percent (97.2%) of the patients were diagnosed with appendicitis on histopathology. The combined accuracy, sensitivity, specificity, PPV, and NPV for TLC, neutrophil count, and ultrasonography came out to be 94.71%, 97.18%, 82.14%, 96.50%, and 85.19%, respectively, as shown in Table 3. The combined tests were evaluated by the cross-tabulation method. The area under the curve for combined TLC, neutrophil count, and ultrasound was found to be 0.897 (refer to Table 3 and Figure 5).

## Discussion

Acute appendicitis is diagnosed clinically in less than 50% of cases [12]. In many cases, the symptoms are atypical or equivocal and require lab investigations or imaging modalities to support or rule out the diagnosis. Nevertheless, even with the help of these diagnostic tools, the rate of perforation of appendices and the rate of negative laparotomy remains high [13]. Authors in various studies have found that ultrasound and CT have high sensitivity and specificity for atypical and equivocal cases of acute appendicitis. However, these investigations are expensive, have radiation hazards, are operator-dependent, and need a radiologist to interpret findings, especially in equivocal cases. This has created a need to find the right combination of blood markers and radiological investigations; that will help surgeons with limited resources in developing countries to accurately diagnose patients [14].

TLC is a commonly used supportive marker for diagnosis and is reported to be > 11,000/mm^3^ in 80%-90% of cases of acute appendicitis. In complicated cases, such as gangrene or perforation, it may be raised up to 18,000/mm^3 ^[15]. Similarly, the results of our study showed that TLC was greater than 11,000/mm^3^ in 83.1% of patients with histopathological evidence of acute appendicitis. In our study, the accuracy of TLC > 11,000/mm^3 ^(as a diagnostic tool) was found to be 82.94%, with sensitivity and specificity being 83.10% and 82.14%, respectively.

Another inflammatory marker, frequently employed for diagnosis, is neutrophil count, which is reported to be greater than 75% (left shift) in 80%-90% of cases [16]. In our study, 87.8% of patients having a neutrophil count greater than 75% were true positive, confirmed on histopathological report findings. The accuracy of the neutrophil percentage was calculated to be 88.82%, sensitivity of 88.03%, and specificity of 92.86%. These recorded results validate the observations made by studies conducted earlier [17-18].

Ultrasound is frequently used as the initial imaging modality for the diagnosis of acute appendicitis, especially in pregnant women, children, and the elderly. However, making an accurate diagnosis in obese patients can prove to be difficult [19]. Modern advancements have made it possible to detect the appendix in up to 97% of cases and the associated inflammatory changes of acute appendicitis, making it a highly sensitive tool. The accuracy of ultrasound in detecting acute appendicitis also depends on the expertise of the radiologist [20]. In our study, 127 out of 132 patients that tested positive on ultrasonography, turned out to be true-positive as confirmed on the histopathological report. The accuracy of U/S was 88.24%, sensitivity was 89.44%, and specificity was 82.14%.

When patients having TLC > 11,000/mm^3^ and neutrophil count > 75% were combined into one group, the combined accuracy was found to be 88.24%, sensitivity was 89.44%, and specificity was 82.14%. Lau et al. observed similar results in their study [18]. Similarly, patients having TLC > 11,000/mm^3^, neutrophil count > 75%, and ultrasound features showing a non-compressible, blind-ending loop and the diameter of the appendix > 7 mm were combined into one group. The combined results showed a sensitivity of 97.18%, specificity of 82.14%, PPV of 96.50%, and NPV of 85.19%. These results are consistent with studies conducted previously and favor performing routine TLC, neutrophil count, and U/S in patients clinically suspected of having acute appendicitis [21-24]. The NPV for combined TLC and the neutrophil count was 60.53%; however, when U/S was combined with these two investigations, the NPV was 85.19%. Hence, acute appendicitis may be ruled out when these two blood investigations are within the normal range and U/S findings do not show acute inflammatory changes in the appendix and surrounding structures.

The limitations of our study were that it was a single-center study and cannot be termed as truly representative of the entire population so the results cannot be generalized to the whole population. Also, different radiologists performed U/S on patients, depending on availability and working shifts, which could have lead to a wide variation in diagnostic accuracy.

## Conclusions

Patients clinically suspected of having acute appendicitis should be considered for combined TLC, neutrophil count, and U/S for confirmation. Although TLC and neutrophil count individually are not specific for the diagnosis of acute appendicitis and may be raised in other diseases, the combined results of all three investigations yield high accuracy, sensitivity, specificity, PPV, and NPV. A CT scan should be reserved for patients in which acute appendicitis cannot be ruled out with the results of these combined investigations.
